# Landscapes of Biochemical Warfare: Spatial Self-Organization Woven from Allelopathic Interactions

**DOI:** 10.3390/life13020512

**Published:** 2023-02-13

**Authors:** Sylvestre Carvalho, Henrique Mota, Marcelo Martins

**Affiliations:** 1 Institute for Advanced Studies, University of São Paulo, São Paulo 05508-050, Brazil; 2 Department of Physics, CFisUC, Center of Physics, University of Coimbra, 3004-516 Coimbra, Portugal; 3 Department of Physics, Federal University of Viçosa, Viçosa 36570-900, Brazil; 4 National Institute of Science and Technology for Complex Systems, Centro Brasileiro de Pesquisas Físicas, Rio de Janeiro 22290-180, Brazil; 5 Ibitipoca Institute of Physics (IbitiPhys), Conceição do Ibitipoca 36140-000, Brazil

**Keywords:** pattern formation, rock–paper–scissors, cyclic interactions, biodiversity, Monte Carlo simulation

## Abstract

Evidence shows that diversity and spatial distributions of biological communities are largely driven by the race of living organisms in their adaptation to chemicals synthesized by their neighbors. In this report, the emergence of mathematical models on pure spatial self-organization induced by biochemical suppression (allelopathy) and competition between species were investigated through numerical analysis. For both random and patched initial spatial distributions of species, we demonstrate that warfare survivors are self-organized on the landscape in Turing-like patterns driven by diffusive instabilities of allelochemicals. These patterns are simple; either all species coexist at low diffusion rates or are massively extinct, except for a few at high diffusivities, but they are complex and biodiversity-sustained at intermediate diffusion rates. “Defensive alliances” and ecotones seem to be basic mechanisms that sustain great biodiversity in our hybrid cellular automata model. Moreover, species coexistence and extinction exhibit multi-stationarity.

## 1. Introduction

Biochemical warfare sustained by the synthesis and release of toxic chemical compounds is a pervasive phenomenon in biological communities, ranging from microorganisms to plants. In bacteria [1], yeasts [2], and other fungi [3], warfare weapons are antibiotic compounds that kill or inhibit the growth of sensitive strains from their own genotypes or different species. In plants, frequently, biochemical weapons are secondary-metabolic phytotoxins, which suppress the germination or growth of neighboring plants [4]. Amazingly, even distinct populations of animal cells, normal or malignant, fight the biochemical war along cancer progression. For instance, glycolytic carcinoma cells excrete large amounts of lactic acids toxic to the surrounding normal cells. The ensuing tissue acidification stimulates tumor growth and invasion [5]. In the most aggressive brain tumor, glioblastoma, glioma cells secrete ATP into the microenvironment. The extracellular ATP itself has low cytotoxic effects on normal cells. However, the degradation of this ATP in adenosine by ectonucleotidases overexpressed on the membranes of glioma cells induces widespread apoptosis among adjacent normal cells [6]. Hence, the race of microbes, plants, and animal cells (normal and malignant) to adapt to the chemicals secreted by their neighbors may regulate species coexistence, shape community composition, and drive the spatial distributions of living organisms in their habitats.

From a pragmatic viewpoint, the importance of these biochemical weapons is immense. For instance, allelochemicals are exploited by several invasive plants to disrupt inherent–co-evolved interactions among long-associated native species, paving the road for the invasion of foreign communities [7]. These biological invasions represent major threats to the functioning of ecosystems, biodiversity conservation, water availability, and agricultural production worldwide [8,9,10,11]. Therefore, preventing and predicting spreading patterns of biological invasion emerge as imperative tasks in an ecologically sustained world. Nevertheless, large-scale observations in the nature of spatial allelopathic patterns are still scarce.

From a theoretical viewpoint, the arms race between living organisms is at the core of fundamental issues shared by diverse fields. Indeed, biological invasions are interesting examples of instability, spontaneous symmetry breaking, and pattern formations in complex systems. The invasion of habitats by alien species can progress along different paths. Invasions frequently unfold through smooth stationary traveling population waves in homogeneous environments [12,13,14]. The invasions of heterogeneous environments (or under the influence of other species) can generate transient or oscillatory traveling fronts before the formation of stationary spatial patterns [15,16]. Concerning the self-assembly of biological communities, understanding how the arms race (involving several species) regulates the pattern formation processes, leading to the coexistence of various species, is a big challenge for ecological and evolutionary biology [17]. Naively, the ubiquity of biochemical warfare and resource competition between species imposes, paradoxically, severe constraints on biodiversity.

Recently, we proposed and investigated an eco-evolutionary model based on ordinary differential equation(s) (ODE) for community assembly [18,19]. In this model, species interact through competition (intra- and interspecific) and allelopathic suppression. It was found that in a homogeneous environment, species-rich communities can only be assembled in the context of weak biochemical warfare between organisms, and even under this regime, species only interact with a few others. Moreover, successive invasion events generate species interaction networks, exhibiting Gaussian and Weibull distributions at weak and strong allelopathy, respectively. Limited biodiversity is an emerging scenario; hence, we hypothesize that biodiversity must increase in spatially extended landscapes. In the present paper, we analyzed a partial differential equation (PDE)-based model (i.e., a spatially explicit version of our ODE model), hybrid cellular automata (CA), and an agent-based model for allelopathic warfare involving species that also compete for common resources. Our main goal was to evaluate the effects of spatial self-organization on biodiversity. Thus, employing numerical integration and simulations, we focused on the spatial patterns generated by the population dynamics of several allelopathic-interacting species.

## 2. Mathematical Models for the Biochemical Warfare between Species

In this section, two mathematical approaches designed for allelopathic warfare and resource competition between several species, which provide explicit geographical information, are presented. They include deterministic PDE and stochastic hybrid CA models.

### 2.1. The PDE-Based Model

The arms race, involving *l* living organisms, is described by the following system of coupled–dimensionless PDEs:

(1) $$\begin{array}{ccc} \partial_{t}N_{i} & = & d_{i}\nabla^{2}N_{i}+r_{i}N_{i}\left(1-\sum_{j=1}^{l}\nu_{ij}N_{j}\right)-\sum_{j\neq i}^{l}\mu_{ij}\Phi_{ij}\left(B_{j}\right)N_{i}\\ \partial_{t}B_{i} & = & D_{i}\nabla^{2}B_{i}+\beta_{i}N_{i}-\delta_{i}B_{i}-\sum_{j\neq i}^{l}\gamma_{ij}N_{j}B_{i} \end{array}$$

where **N** $=(N_{1},N_{2},\ldots ,N_{l})$ and **B** $=(B_{1},B_{2},\ldots ,B_{l})$ are, respectively, the species population densities and their secreted toxin concentrations. So, the species replicate at growth rates $r_{i}$ spread into space and interact with other species. These interactions occur via intra- and interspecific resource competition, as well as biochemical suppression mediated by synthesized and released toxic secondary chemical compounds (microcins, phytotoxins, etc.) that enhance the mortality of target species. The parameters $\nu_{ij}$, controlling the strengths of intra- and interspecific resource competitions, are randomly chosen in the range $0<\nu_{ij}<1$, ∀i≠j. In turn, $\nu_{ii}=1\;\forall i$. Biochemical suppression is specified by allelochemical networks defined by different sets of coupling constants $\mu_{ij}=\mu \xi_{ij}$, where

$$\begin{array}{c} \xi_{ij}=\left\{\begin{array}{cc} 1, & \;\mathrm{if}\;j\;\mathrm{poisons}\;i\\ 0, & \mathrm{otherwise}. \end{array}\right. \end{array}$$

Allelopathic functional responses of the Holling type I

(2) $$\begin{array}{c} \Phi_{ij}^{\left(k\right)}=\left\{\begin{array}{cc} B_{j} & \;\mathrm{if}\;k=1\\ \gamma_{ij}\;N_{i}\;B_{j} & \;\mathrm{if}\;k=2 \end{array}\right. \end{array}$$

describe species *i* mortality induced by the free or uptaken allelochemical $B_{j}$. The k=1 functional response depends on the local, free toxin concentration, whereas the k=2 response is a function of the locally uptaken allelochemical amount. The toxin’s uptake, secretion, and natural degradation rates are, respectively, $\gamma_{i,j}=\gamma \xi_{ij}$, $\beta_{i}$, and $\delta_{i}$. Every species and its corresponding toxin spread throughout the environment according to normal diffusion processes characterized by diffusivities $d_{i}$ and $D_{i}$, respectively.

The PDE system (1) was numerically integrated using a finite difference method [20,21] and null Dirichlet boundary conditions at the edges of a two-dimensional landscape of a linear size *L*. So, $N_{i}\left(x,y,t\right)=B_{i}\left(x,y,t\right)=0$ if x,y=0 or *L*∀i. Two different initial conditions were tested. In the first one, species were distributed on circular patches spatially isolated from any other patch. The initial population densities and toxin concentrations at patch *i* are $N_{i}\left(0\right)=0.25$, $N_{i\neq j}\left(0\right)=0$, and $B_{i}\left(0\right)=0$∀i. Outside of the patches, the landscape is free from species and toxins. In the second initial condition, the landscape is partitioned as a grid of small square patches, with a linear size of a≪L, and each patch *k* is occupied by only one randomly chosen species *i*. The initial density $N_{i}\left(0\right)$ of the chosen species is a random value selected in the range [0.01,1], but $N_{j}\left(0\right)=0$∀j≠i on patch *k*. Again, $B_{i}\left(0\right)=0$∀i is everywhere on the landscape.

### 2.2. The Hybrid CA-Model

In order to avoid some natural difficulties introduced by the continuous character of a PDE-based model, such as the coexistence of various non-vanishing population densities at the same spatial point or the need to set up arbitrary local thresholds for species extinction, a hybrid agent-based framework retaining the basic traits of the previously described model was considered. In this version, the discrete nature of the agents and the spatial exclusion rule that forbids the simultaneous occupation of a site by two or more agents circumvent the aforementioned difficulties and also provide a greater biological appeal. Moreover, in a hybrid agent-based model, species interactions were implemented at the individual level via a set of mechanistic action rules adapted from reference [22], as discussed below.

The environment is a square lattice of L×L sites, which can be empty or occupied by a single individual. Biologically, each site represents, for example, a patch with a size scale comparable to those of a plant’s rhizosphere. Periodic boundary conditions are used. Each organism is a cellular automaton (CA) [23,24] or simply an individual agent. The competition primarily operates on the individual, affecting its replication, survival, and dispersal. At each time step, *N* agents are randomly selected and can replicate or die with equal probability according to the following rules:

**Replication**. A species *k* individual can replicate with probability $p_{rep}^{k}$ if the von Neumann neighborhood has at least one empty site. Its descendent will randomly occupy one of those empty sites. The replication probability is

(3) $$p_{rep}^{k}\left(\left\{B_{l\neq k}\left(i,j,t\right)\right\}\right)=p_{0}^{k}\left[1-\frac{{\left(\sum_{l\neq k}\xi_{l,k}B_{l}\right)}^{2}}{C_{k}+{\left(\sum_{l\neq k}\xi_{l,k}B_{l}\right)}^{2}}\right],$$

where $p_{0}^{k}$ is the *k*-species’ natural replication probability in the absence of biochemical warfare, $B_{l\neq k}\left(x,t\right)$ is the concentration, on-site (i,j) at time *t*, of the allelochemical secreted by the species *l*, and $C_{k}$ is a model parameter related to the *k*-species resistance to chemical poisoning. Again, $\xi_{l,k}=1$ if species *l* poisons species *k* and $\xi_{l,k}=0$ otherwise.

Finally, when two or more distinct species try to disperse their progenies to the same empty site simultaneously, this site will be colonized by an individual of that species exhibiting the largest local $p_{rep}^{k}$.

**Death**. A *k*-species individual occupying the site (i,j) can die with a probability $p_{del}^{k}$ given by

(4) $$p_{del}^{k}\left(\left\{B_{l\neq k}\left(i,j,t\right)\right\}\right)=q_{0}^{k}\left[\frac{{\left(\sum_{l\neq k}\xi_{l,k}B_{l}\right)}^{2}}{A_{k}+{\left(\sum_{l\neq k}\xi_{l,k}B_{l}\right)}^{2}}\right],$$

where $q_{0}^{k}$ is the *k*-species natural death probability in isolation, $B_{l\neq k}\left(x,t\right)$ is the allelochemical concentration on site (i,j) at the time *t* produced by the species *l*, and $A_{k}$ sets the species *k* resistant to the death induced by chemical poisoning. Again, $\xi_{l,k}=1$ if species *l* poisons species *k* and $\xi_{l,k}=0$ otherwise. Naturally, the death of an individual generates an empty site.

**Toxin dispersion**. Every individual of each species engaged in the biochemical warfare secrete toxins that disperse throughout the landscape according to a normal diffusion process described by the PDE:

(5) $$\partial_{t}B_{k}=D_{k}\nabla^{2}B_{k}+\beta_{k}\sum_{x_{i}^{k}}\delta \left(x-x_{i}^{k}\right)-\gamma_{k}B_{k},$$

where $D_{k}$, $\beta_{k}$, and $\gamma_{k}$ are, respectively, the diffusivity, synthesis, and natural degradation rates of the *k*-species toxin. This equation assumes that the sources of toxin *k* are individuals of the species *k* located at the lattice sites corresponding to the positions $x_{i}^{k}$.

The hybrid agent-based model simulations were implemented through the following procedure. Initially, the species are distributed on the square lattice. Two initial conditions, namely, random dispersion or isolated localized square lattice with L=100 adapted from the previous subsection, were used. At each time step, all N(t) individuals are simultaneously selected. Each selected agent can die with probability $p_{del}^{k}$ or replicate with probability $p_{rep}^{k}$, and colonize one of their empty nearest-neighbor sites if its local replication probability is the largest one. We updated the species distributions on the lattice; the new spatial profiles of toxin concentrations were determined according to Equation (5), which is relaxed by 400 iterations (quasi-stationary solution). At the end of this sequence of actions, a new Monte Carlo time step (MCS) begins and the entire procedure is iterated.

## 3. Numerical and Simulation Results

### 3.1. Continuous, Deterministic Population Dynamics

**Figure 1:**
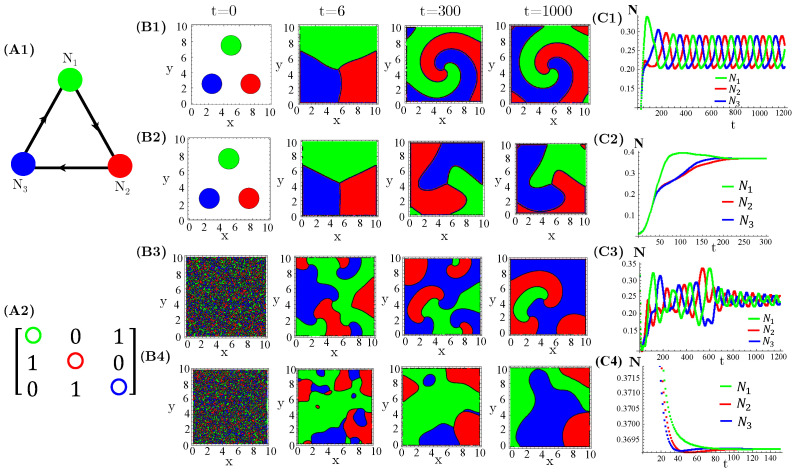
Evolution in time of population densities $N_{1}$, $N_{2}$, and $N_{3}$ for three species engaged in a RPS game. (**A1**) The cyclic allelopathic interaction network and (**A2**) its corresponding adjacency matrix $\xi_{ij}$. The color corresponding to species are defined in (**A1**). (**B1**–**B4**) Spatial patterns generated at four different times starting from patched (**B1**,**B2**) or random (**B3**,**B4**) initial conditions. The color assigned to each spatial point is that of the locally dominant species, i.e., the one sustaining the greatest density. (**C1**–**C4**) Species-spatial average densities as functions of time corresponding to scenarios (**B1**–**B4**). The model parameters are fixed in $r_{i}=0.3$, $\nu_{i}=0.5$, $\mu_{i}=0.4$, $\beta_{i}=0.5$, $\delta_{i}=0.1$, $\gamma_{i}=0.1$, and $D_{i}=d_{i}=0.005$∀*i*. The functional responses to toxins used were $\Phi^{\left(1\right)}$ (**B1**,**C1**,**B3**,**C3**) and $\Phi^{\left(2\right)}$ (**B2**,**C2**,**B4**,**C4**).

The case of N=2 interacting species, extensively investigated in reference [14], exhibits either simple homogeneous or striped spatial patterns for one species extinction or coexistence, respectively. Moreover, species extinction occurs through progressive invasion waves of the survival species. Concerning N=3 species, firstly, we considered three allelopathic populations interacting through a rock–paper–scissors (RPS) game (Figure 1). For patched initial conditions, species are spatially self-organized in spiral patterns after collisions of the expanding occupied circles (Figure 1(B1,B2)). In turn, the initial random and uniform distributions of species destroy the spirals and generate regular (Figure 1(B3)) or irregular (Figure 1(B4)) concentric patterns. Furthermore, enhanced allelopathic suppression through the use of functional response $\Phi^{\left(1\right)}$ reinforces species spatial self-organization in spiral or concentric patterns. Indeed, for instance, the spirals (Figure 1(B1)) are sharper than those exhibited by functional responses $\Phi^{\left(2\right)}$ (Figure 1(B2)). The same is true for the concentric patterns (Figure 1(B3,B4)). Moreover, enhanced allelopathy generates lower average population densities oscillating in time (Figure 1(C1,C3)), whereas attenuated suppression only induced by absorbed toxins ($\Phi^{\left(2\right)}$ functional responses) leads to higher and fixed stationary densities (Figure 1(C2,C4)). As can be noticed, random initial conditions produce longer and irregular transients to reach the limit of cycle attractors (Figure 1(C1,C3)).

**Figure 2:**
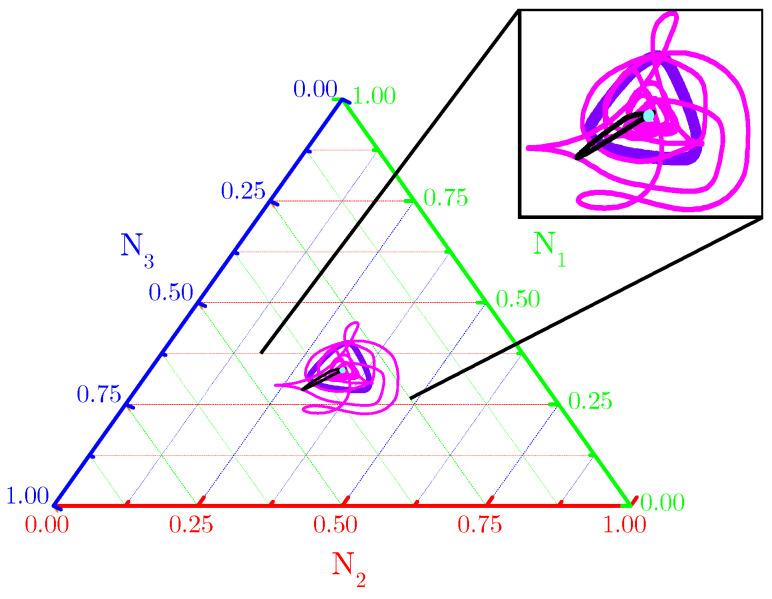
Ternary diagram for orbits ($\langle N_{1}\left(t\right)\rangle ,\langle N_{2}\left(t\right)\rangle ,\langle N_{3}\left(t\right)\rangle )$ described by spatially averaged fractional densities of the three species. Purple and black orbits are typical for functional responses $\Phi^{\left(1\right)}$ (enhanced mortality) and, respectively, patched or random initial conditions, scenarios in Figure 1(B1,C1,B3,C3); pink and cyan orbits are typical for functional responses $\Phi^{\left(2\right)}$ (attenuated allelopathic suppression) and, respectively, patched or random initial conditions, i.e., scenarios in Figure 1(B2,C2,B4,C4). Model parameter values are those listed in Figure 1 and all orbits are integrated until $t=1.2\times 10^{3}$.

**Figure A1:**
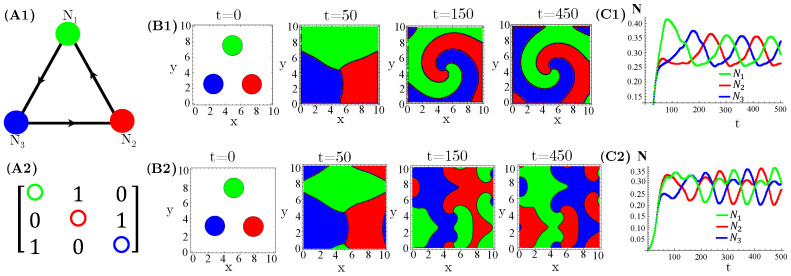
Graph (**A1**) and their matrix (**A2**) associated to rock–paper–scissors (RPS) population dynamics (spatial patterns and average species densities) generated by patched initial conditions, enhanced allelopathy (toxin functional response $\Phi^{\left(1\right)}$), and Newman (upper panels **B1**,**C1**) or periodic boundary conditions (lower panels **B2**,**C2**). The model parameter values are those used in Figure 1.

The ternary diagram (Figure 2) shows evidence that single orbits ($\langle N_{1}\left(t\right)\rangle ,\langle N_{2}\left(t\right)\rangle ,\langle N_{3}\left(t\right)\rangle )$ for spatially averaged fractional densities (based on initial conditions and subjected to the toxin’s functional responses considered in Figure 1) are really attracted to fixed points and limit cycles. Our numerical results indicate that spiral or concentric nontrivial spatial patterns self-organize from the RPS suppression games in landscapes subjected to periodic and Newman boundary conditions (Figure A1—Appendix A).

**Figure 3:**
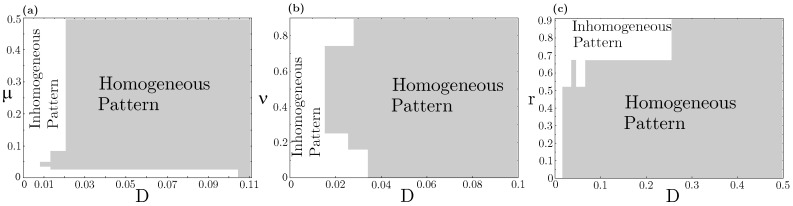
RPS pattern formation “phase diagram”. The white color indicates an inhomogeneous (e.g., spiral or concentric) spatial pattern, whereas the gray color represents a homogeneous pattern for the survivor species at the stationary state. Values β=0.5, δ=0.1, and γ=0.1 are fixed. Different sections of the model parameter space were tested: (**a**) r=0.3 and ν=0.5; (**b**) r=0.3 and μ=0.05; (**c**) ν=0.5 and μ=0.05. Equation (1) was numerically integrated up to t=3000 from patched initial conditions shown in Figure 1(A1).

**Figure A2:**
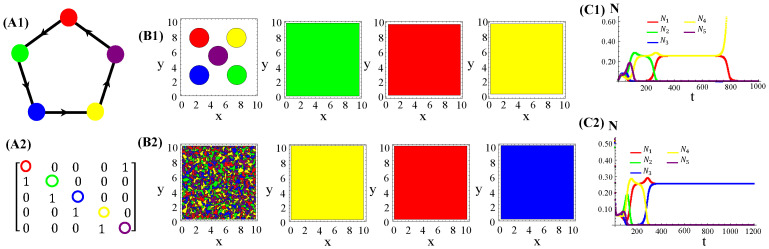
Five species competing for resources and with cyclic allelochemical suppressions. (**A1**) Schematic illustration of five-species allelochemical warfare. Arrows point from suppressor to suppressed (**A2**). The allelochemical interaction matrix associated to the graph in (**A1**). The initial populations are spatially distributed either regularly in single, disjunct, isolated, and circular patches (**B1**) or randomly (**B2**) in adjacent. The corresponding spatial distributions of the species at three distinct times are shown for each initial condition. The different colors indicate the locally dominant species. The evolution in time of the population densities are shown in (**C1**,**C2**). The results refer to response functions dependent on the total local concentration of allelochemicals. Their values were fixed in D=d=0.5 (high diffusivities); the rest of the model parameter values are those used in Figure 1.

Concerning species coexistence, even at the strong interspecific competition ($\nu_{ij}>1\;\forall i\neq j$), the N=3 RPS allelopathic game can generate stationary states in which all three species coexist in spatially extended landscapes. This scenario is impossible in a spatially implicit approach based on ordinary differential equations (ODEs), which invariably leads to species extinction, except the stronger one accounts for both resource competition and allelochemical suppression. Nevertheless, in our model, species coexistence spatially self-organized in nontrivial patterns (spirals and concentric, but non-homogeneous patterns) is primarily determined by species and toxin diffusivities. We analyzed three distinct sections of the model parameter space, namely, μ×D, ν×D, and r×D planes (Figure 3). Each trait μ (mortality rate induced by the toxin), ν (competition pressure upon common resources), *r* (species replication rate), and $D=D_{i}=d_{i}$ (species and its toxin diffusivities) were assumed the same for every three species. As one can see, all species coexistence and self-organizing spatial dynamics are observed in very constrained regions of the parameter space. A general rule emerges: large diffusivities of species and their toxins trigger extinction and lead to the homogeneous dispersion of the survival species on the landscape (Figure A2—Appendix B). In contrast, low diffusivities allow species coexistence and self-organizing non-trivial spatial dynamics. On the vertical axis $D=D_{i}=d_{i}=0$, only homogeneous spatial patterns are observed since in dynamical Equation (1) ODEs are reduced in the process involving both resource competition and allelochemical suppression.

**Figure A3:**
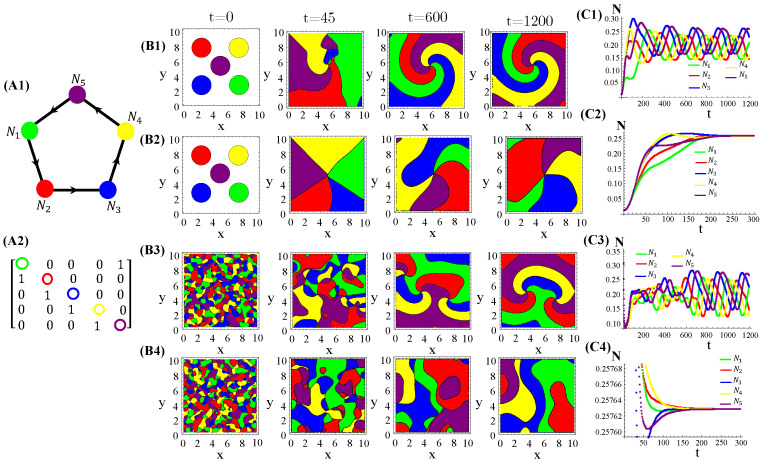
Five species competing for resources and with cyclic allelochemical suppressions. (**A1**) Schematic illustration of five-species allelochemical warfare. Arrows point from suppressor to suppressed (**A2**). The allelochemical interaction matrix associated to the graph in (**A1**). The initial populations are spatially distributed either regularly in single, disjunct, isolated, and circular patches (**B1**,**B2**) or randomly in adjacent, disjunct but disordered patches (**B3**,**B4**). The corresponding spatial distributions of the species at three distinct times are shown for each initial condition. The different colors indicate the locally dominant species. The evolution in time of the population densities are shown in (**C1**–**C4**). The results in (**B1**,**C1**) and (**B3**,**C3**) refer to response functions dependent on the local concentration of allelochemicals, whereas those in (**B2**,**C2**) and (**B4**,**C4**) are for response only to locally uptaken toxins. The competition and allelochemical traits are the same for all species. The paprameter used are the same as in Figure 1.

**Figure A5:**
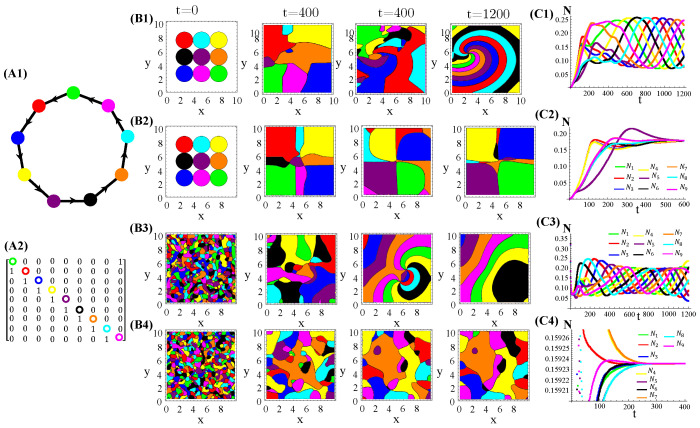
Nine species competing for resources and with cyclic allelochemical suppressions. (**A1**) Schematic illustration of five-species allelochemical warfare. Arrows point from suppressor to suppressed (**A2**). The allelochemical interaction matrix associated to the graph in (**A1**). The initial populations are spatially distributed either regularly in single, disjunct, isolated, and circular patches (**B1**,**B2**) or randomly in adjacent, disjunct but disordered patches (**B3**,**B4**). The corresponding spatial distributions of the species at three distinct times are shown for each initial condition. The different colors indicate the locally dominant species. The evolution in time of the population densities are shown in (**C1**–**C4**). The results in (**B1**,**C1**) and (**B3**,**C3**) refer to response functions dependent on the local concentration of allelochemicals, whereas those in (**B2**,**C2**) and (**B4**,**C4**) are for response only to locally uptaken toxins. The competition and allelochemical traits are the same for all species. The paprameter used are the same as in Figure 1.

**Figure A6:**
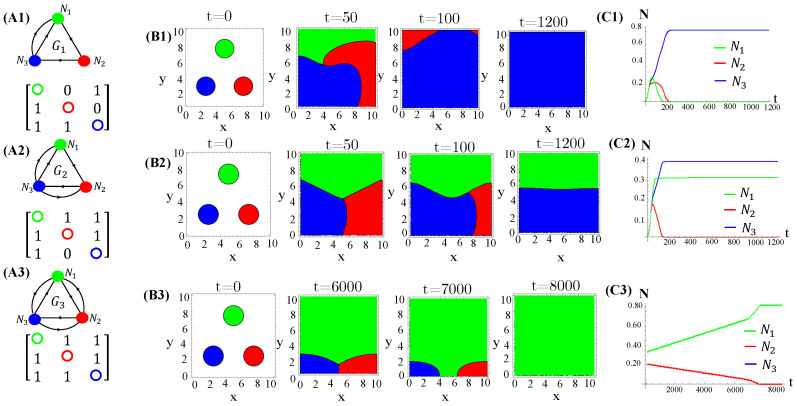
Graphs and their matrices (**A1**–**A3**) associate to population dynamics (spatial patterns (**B1**–**B3**) and average species densities (**C1**–**C3**)) generated by patched initial conditions under enhanced allelopathy (functional response $\Phi^{\left(1\right)}$) and distinct allelochemical networks. The model parameter values are those used in Figure 1.

**Figure A7:**
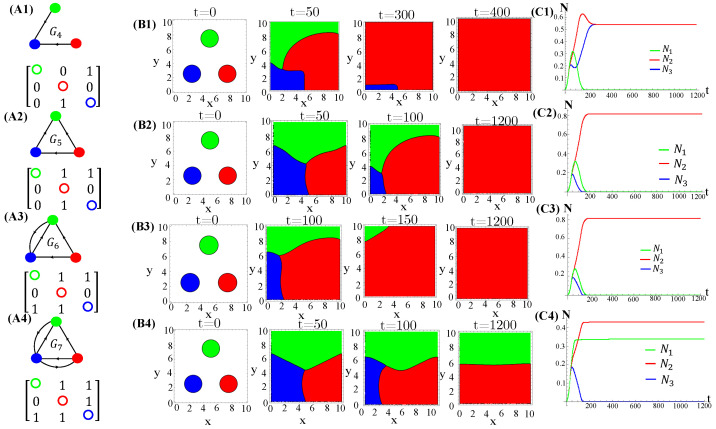
Graphs and their matrices (**A1**–**A4**) associate to population dynamics (spatial patterns (**B1**–**B4**) and average species densities (**C1**–**C4**)) generated by patched initial conditions under enhanced allelopathy (functional response $\Phi^{\left(1\right)}$) and distinct allelochemical networks. The model parameter values are those used in Figure 1. This is a continuation of Figure A6.

**Figure A8:**
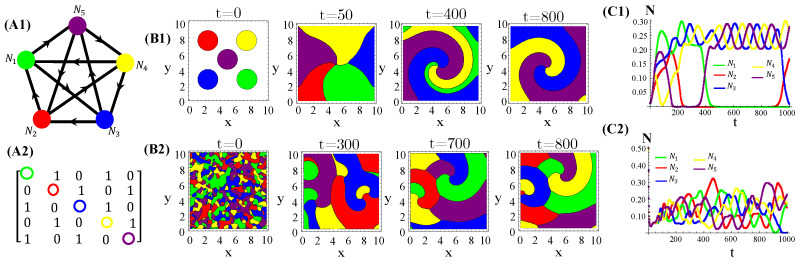
The same as in Figure A3. In addition to cyclic allelochemical suppression, one is suppressed by the others of the two next nearest neighbor species (**A1**). (**A2**) describes the adjacency matrix. Spatial distribution of species generated by patched (**B1**) and random (**B2**) initial conditions at distinct times. In (**C1**,**C2**), the extinction of, respectively, one and two species occurs. The data refer to the response functions $\Phi^{\left(1\right)}$. The model parameter values are those used in Figure 1.

Secondly, we also tested cyclic allelopathic networks involving more than N=3 species. Appendix C reports our major findings. Results for spatial patterns and temporal evolution of species populations in ecosystems with N=5,7,and 9 species engaged in cyclic games are illustrated (Figure A3–Figure A5). They are similar to those illustrated in Figure 1 for the N=3 RPS game. In addition, successive extinction events unfold cascades of changes in the species dominance of organisms and their toxins are highly diffusive (Figure A2—Appendix B). Moreover, the current dominant species promptly spread homogeneously on the landscape, but soon it will be extinct (if not the ultimate survivor of the allelochemical warfare). Since in cyclic networks of species endowing equal ecological and biochemical traits, the ultimate survivor is exclusively determined by the initial conditions and the toxin’s network, spatially extended cyclic games exhibit multistability, a hallmark of the dissipative structure. Finally, non-cyclic suppression networks were tested and some results are reported (Figures—Appendix D). The key feature is that spatially striped patterns unfold if two species survive allelochemical warfare (Figure A6–Figure A8). Particularly, each stripe is dominated by a single survivor or two survivors and, again, multistability is present (Figure A6 and Figure A7). The cyclic game combined with star interactions can generate the extinction of at least two species over a time interval (Figure A8). Extinct species may reappear, leading other species to extinction. In the case of allelochemical parameters being fixed, the warfare’s outcomes will depend on the way toxins affect the species. If the local amount of a toxin inhibits its target, the stationary state is either a limit cycle or stable focus. In turn, if only the locally uptaken toxin affects its target, the time evolution is driven to a fixed point.

### 3.2. Hybrid and Stochastic CA Dynamics

**Figure 4:**
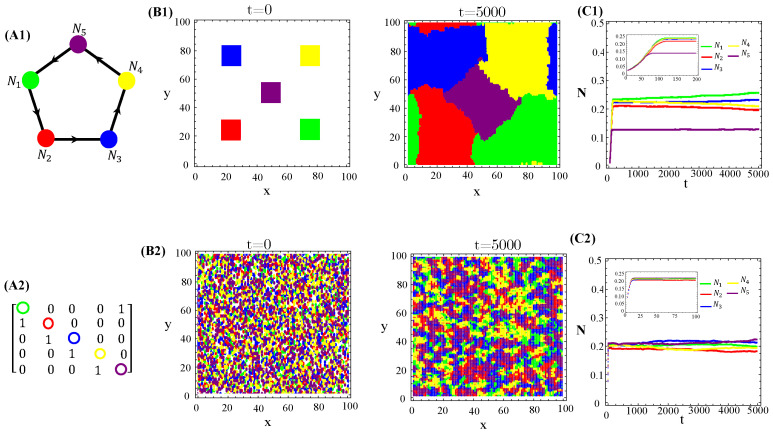
Graph (**A1**), the adjacency matrix of allelopathic interactions (**A2**), the initial distribution of species and their long-term spatial patterns (**B1**,**B2**), temporal evolution of density populations (**C1**,**C2**) for the N=5 RPSLS suppression game starting from patched (top) or random (bottom) conditions. The CA parameter values are L=100, $p_{rep}^{0,k}=p_{del}^{0,k}=0.5$, $A_{k}=C_{k}=0.5$, $\beta_{k}=0.7$, $\gamma_{k}=0.2$, and $D_{k}=0.001$ for k=1,…,5.

**Figure A9:**
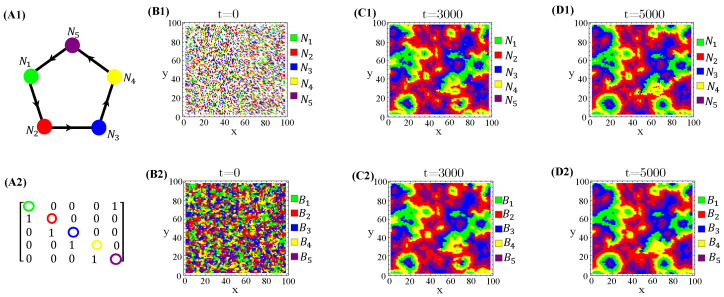
Spatialdistribution snapshots of species (**B1**–**D1**) and their toxin concentrations (**B2**–**D2**) at three distinct times. The allelopathic network (**A1**), correspondent adjacency matrix (**A2**), and parameter values are the same ones used in Figure 5.

The population dynamics and their spatial patterns generated by the N=5 hybrid CA (Figure 4) are counterparts to the RPS suppression game (Figure A3). At low toxin diffusivities, species initially distributed in patches on the landscape remain patched along the time evolution. Thus, the spiral patterns observed (Figure A3(B1)) and temporal oscillations in species populations (Figure A3(C1)) are destroyed. The CA version of the RPS game leads to a stationary fixed point in which all five species coexist, instead of a limit cycle. Similarly, spatial random distributions of a species are sustained under the CA rock-paper–scissor–lizard–Spock (RPSLS) game dynamics and, therefore, concentric patterns do not unfold (Figure A3(C3)). The absence of both spiral and concentric spatial patterns and periodic oscillations in species populations at low diffusivity regimes is due to a key ingredient, namely, the spatial exclusion rule assumed in the CA version of the continuous model. Since only one specimen can occupy a single (formerly empty) lattice site and toxins diffuse significantly over a small characteristic length scale $l_{c}∼\sqrt{D/\gamma }$, species spreading on the landscape is strongly impaired. Consequently, the nature of the initial spatial pattern tends to be preserved along the ecosystem evolution in time. Moreover, our results demonstrate that species and their released toxins are spatially correlated (Figure A9—Appendix E).

**Figure 5:**
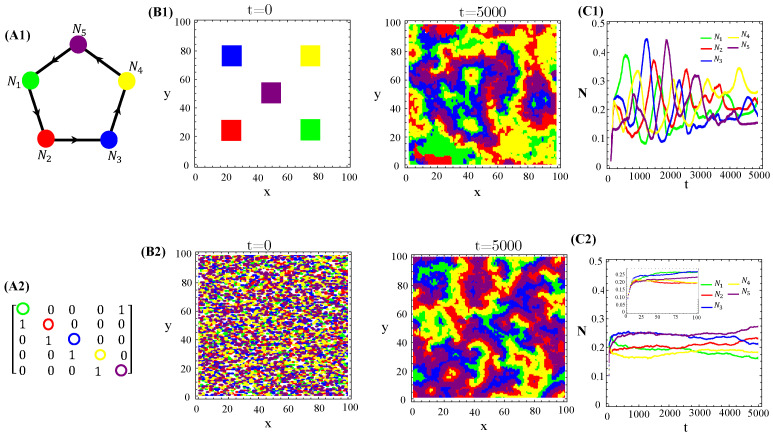
Graph (**A1**), adjacency matrix of allelopathic interactions (**A2**), initial distribution of species and long-term spatial patterns (**B1**,**B2**), temporal evolution of density species populations (**C1**,**C2**) for the N=5 RPSLS suppression game starting from the patched (top) or random (bottom) conditions. The toxin diffusion constants are (**B1**) D=0.3 and (**B2**) D=0.5. The other CA parameter values are the same as used in Figure 4.

Nevertheless, higher toxin diffusivities drastically change the previous scenario. Indeed, the stationary spatial patterns unfolded by the RPSLS allelochemical game become irregular mixtures of fragmented spots and plumes sometimes reminiscent of concentric and spiral patterns (Figure 5). So, the memory of initial species dispersion on the landscape is lost in the long term and similar spatial distributions of species are always generated asymptotically. Moreover, biological populations fluctuate in time and such fluctuations can be large and highly irregular depending on toxin diffusivities (Figure 5(C1)).

**Figure 6:**
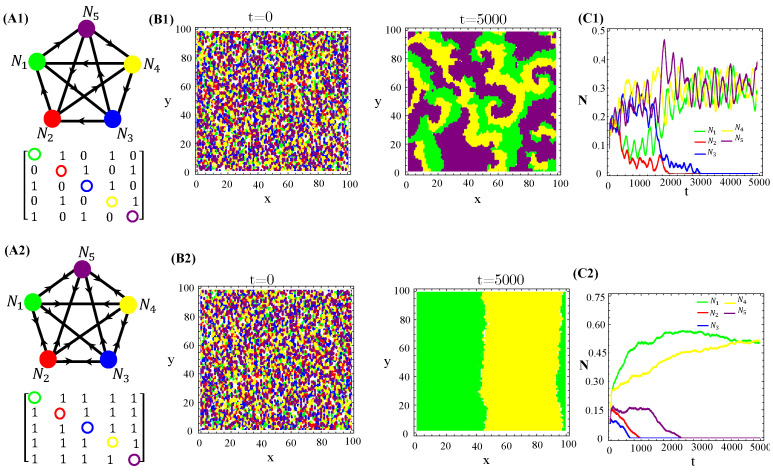
Two and three species extinction events in N=5 non-RPSLS games. (**A1**,**A2**) allelopathic suppression networks, and their associated adjacency matrices. (**B1**,**B2**) Spatial patterns emerging from random initial distributions of species on the landscape. (**C1**,**C2**) Temporal evolution of total species populations. Toxin diffusivities were fixed in D=0.4 (top) and D=0.8 (bottom). The other CA parameter values are those used in Figure 4. The three survivors of biochemical warfare evolved in a cyclic (RPS) game.

We also tested non-RPS-like structures for allelochemical interaction networks. In contrast with the general rule—all species coexistence—valid for N=3,5,and7 “equally armed” competitors engaged in RPSLS (cyclic) games, allelopathic suppression frequently leads to species extinction in non-cyclic networks if toxin diffusivities are not very low. For instance, the extinction (of two or three species) in N=5 non-RPS games is shown (Figure 6). With the emergence of landscapes of spatially striped patterns, each stripe is occupied by only one survivor of the biochemical warfare (Figure 6(B2)). In addition to toxin diffusion capacities, extinction events are also dependent on initial conditions, mainly the topology (structure) of the allelopathic network. As observed for the continuous version, in Equation (1), multistability occurs in the hybrid CA model proposed here when the species are endowed with equal biochemical arms and competition traits.

Our proposed continuous model and its hybrid CA version exhibit qualitative similarities concerning species coexistence, multistability associated with extinction events, populations oscillating in time, and spatial self-organization in varied patterns. However, only the latter approach is able to reveal a key feature from a biological standpoint. The discrete nature of every individual agent (in conjunction with a spatial exclusion principle forbidding two agents from simultaneously occupying the same lattice site) lead to the formation of defensive alliances in allelochemical war. Within a large range of toxin diffusivities values, species self-organize spatially in a hierarchical-nested way (Figure 5). This means that two species do not suppress each other, occupy adjacent regions, or one of them is encircled by the other. Such spatial dissipative structures minimize interfaces between regions occupied by mutually poisoning species and, consequently, foster landscape biodiversity. Indeed, at neither too small nor large characteristic diffusion lengths, allelopathic suppression followed by site colonization occur at interfaces between mutually poisoning species. Furthermore, since in our CA model the emergence and entanglement of rotational spiral waves are neatly impaired by discreteness and spatial exclusion, the formation of defensive alliances seems to be the basic dynamical structure supporting coexistence on the landscape.

## 4. Discussion

From a physicist’s standpoint, ecosystems are nonlinear dynamical systems that self-organize into functional stable attractors if left undisturbed. Along such self-organization processes, spatially heterogeneous landscapes, complex networks of energy and resource flows, as well as trophic relationships may emerge, giving rise to astonishing biodiversity [25,26]. So, uncovering and understanding possible driving mechanisms for spatial pattern formation and the assembly of species-rich communities are imperative tasks that connect the self-organization theory and functional ecology. In the present manuscript, we addressed allelopathy [27] as a pattern-forming mechanism. As it is notoriously difficult to identify allelochemicals and quantify their suppressive effects as well as their dispersion scales in real-world ecosystems, our investigation was essentially based on spatially explicit mathematical models.

Our major findings (derived from both continuous and discrete spatially explicit models for species interacting through resource competition and allelopathy) are as follows:

(i) Species self-organize in inhomogeneous spatial patterns reinforced by enhanced allelopathic suppression. Such patterns include spirals, concentric structures, and stripes. In turn, homogeneous patterns emerge if the biochemical warfare triggers a cascade of species extinction, ultimately leading to a single survivor species. These “Turing patterns” are driven by scale-dependent feedback (SDF) mechanisms, namely, the short-range activation of the growth of a species by its secreted toxin coupled to its long-range inhibition due to suppressive effects of allelochemicals released by competing species. In addition, our hybrid CA model revealed that a SDF mechanism can weave irregular spatial patterns even starting from patched (regular) initial conditions. Thus, SDF can also produce irregular spatial patterns, as recently demonstrated by Zhao et al. [28] for salt marshy ecosystems dominated by *Scirpus mariqueter* plants. In this marine ecosystem, the negative feedback, elicited by the wave impacts and associated water turbulence produced by semidiurnal tides, is stochastic.
(ii) The coexistence of all competing species arises for both RPS (or RPSLS)- and non-RPS (RPSLS)-like allelochemical interaction networks, even in a regime of strong resource competition. This is possible only in spatially extended systems through spatial self-organization. Indeed, in a homogeneous mixture scenario, extinction cascades are always observed. However, all species coexistence occurs in a constrained range of organisms and/or toxin diffusivities, because large diffusion rates effectively correspond to a homogeneous mixture. In particular, our hybrid CA model provides a clear portrait of allelochemical-induced spatial self-organization. At very small toxin diffusivities, all species’ coexistence patterns are either patched or random according to the initial conditions. These simple patterns prevail because species are unable to invade regions colonized by other competitors (the spatial exclusion rule). Hence, the initial distribution of species on the landscape is sustained. At intermediate diffusivity values, toxins diffuse significantly up to the characteristic length scale $l=\sqrt{D/\gamma }$. Consequently, the negative feedback produced by an organism beyond such characteristic lengths is not strong enough to kill all of its competitors present in wider neighborhoods. Competing species survive in inter-patch regions and irregular, more complex spatial patterns resembling spiral or concentric forms emerge from this SDF mechanism. Finally, at large diffusivities, allelopathic suppression remains strong enough to extinct susceptible species over great distances in inter-patch regions. A survivor cannot invade distinct domains because it does not poison other survivors. So, species self-organize spatially in striped or homogeneous patterns for, respectively, more than one or a single survivor.
(iii) Both species dominance and extinction neatly demonstrate the presence of multistability in our models. Particularly, in the hybrid CA version, extinction events are undisputed and independent of any arbitrary non-null threshold fixed for local densities of species. Moreover, the exclusion rule that limits every site occupation to (at most) one individual makes invasion fronts and ecotones (interfaces separating domains of distinct ecosystem states) [27] sharply defined. Under multistability, community assembly can be self-organized in many alternative ways. Our results allow for several coexisting species irregularly patterned in space for not-too-great toxin diffusivities. In contrast, at large diffusivities, very few species invade the landscape. Whatever the scenario, the species that either survive or become extinct during the biochemical warfare are selected by chance through small inhomogeneities in the initial conditions and/or fluctuations inherent to stochastic evolution rules. Neatly, the stochasticity of the CA rules for organism replication and death induces randomness in the negative feedback, which enhances irregular patterning. This disturbing mechanism acting on SDF is absent in the continuous deterministic model that, in turn, generates much more regular spatial patterns.
(iv) “Defensive alliances” and ecotones seem to be the basic mechanisms sustaining great self-organized biodiversity on the landscapes in complex spatial patterns. The key ingredients are discreteness (individual agents), spatial exclusion (at most, one agent occupying a lattice site), and moderate (neither too small nor too large) diffusivities of organisms and their toxins. Under such conditions, allelopathic suppression followed by site colonization occur just around interfaces separating regions dominated by species poisoning each other. Within a domain, mutually neutral organisms can frequently coexist and form defensive alliances. Invasion attempts by any suppressive species from among the remaining ones are defeated by one of the alliance members. Outside a domain, near its interface (ecotone), other suppressive species can be established in the “open” areas. Thus, inside each alliance domain, the species are excluded, but several distinct alliance patches partition the landscape and sustain higher biodiversity. Clearly, spatial patterning may enhance species coexistence and diversity subjected to inhibitory SDF dynamics.

Finally, we briefly relate our findings to the current literature on competing associations, community assemblies, and cyclic dominance. Periodic Turing structures driven by SDF mechanisms were observed in many vegetation patterns, including semiarid plants [29], peat bogs [30], and coastal mussel beds [31]. Earlier mathematical models that described such regular Turing patterns were based on a macroscopic framework focused on partial differential equations for population dynamics. However, in deterministic models, the spatial self-organization of species in irregular patterns remains elusive. Hence, theoreticians are heavily invested in agent-based models in which mechanisms of species competition and coexistence are defined at the levels of individual interactions. Then, the three-species cyclic rock–paper–scissors (RPS) model emerged as a paradigm to understand species diversity [32]. One classical population dynamic exhibiting RPS-like competition is the three-morph mating system in the side-blotched lizard [33]. Several continuous, deterministic, lattice, stochastic versions of RPS-like models revealed hallmark results, namely, complex species coexistence patterns constrained to spatially extended systems [32,34,35], which are drastically affected by individual migration. For low mobility values, long-term coexistence is sustained in expanding domains or randomly dispersed small patches comprised of single species. In contrast, at high mobilities, biodiversity is lost and surviving species are self-organized in either spatial domains (stripes) containing mutually neutral species or a uniform distribution of the ultimate survivor. At intermediate motility values, complex patterns constituted by mutually neutral species emerge. All of these survival scenarios were observed in references [34,36], whereas only the scenarios for low and high diffusivities were reported in references [32,35]. Furthermore, these “phases” exhibit multistability and correspond to distinct communities differing in the groups of surviving species and their spatial patterns. Moreover, studies on RPS models reveal the dynamical emergency of new associations among species, called defensive alliances, which essentially define the number of survivors and spatial patterns formed. Our models addressing the population dynamics of species that compete for common resources and interact through allelopathic suppression networks exhibit all of these hallmark results. Allelopathy plays a key role in ecosystems, ranging from colicinogenic strains of *Escherichia coli* [37], invertebrates in coral reefs [38], and plant communities [4], but only recently was it proposed as a SDF mechanism for pattern formation [27]. A fundamental novelty in our model is that allelopathic suppression is explicitly mediated by diffusive toxins secreted by each competing species. This approach allows us to investigate the effects of higher-order interactions in which species can interact no longer through pairwise mechanisms [39]. We are currently implementing this research project.

## 5. Conclusions

The present work integrates theoretical studies that indicate that scale-dependent feedback (SDF) can produce regular as well as irregular spatial patterns. Here, we focused on allelopathy as the main SDF mechanism for patterning formation. Although pairwise allelopathic suppression represents inhibitory interactions, it can maintain complex spatial coexistence patterns, generate new associations among groups of species—defensive alliances—determining how many of them can coexist under a multistability scenario, as well as promote the mobility/diffusivity-dependent selection of species associations and their corresponding patterns in spatially extended landscapes. Defensive alliances and ecotones (interfaces or boundary layers between such species associations) are central mechanisms promoting the routes from coexistence to the extinction of biological species engaged in biochemical warfare (allelopathy). Finally, spatial–self-organized biodiversity in complex patterns was observed in our models even for interaction network topologies distinct from cyclic dominance, indicating that diversity and the stability of ecosystems are robust concerning the details of the cyclic competition. 

## Figures and Tables

**Figure A4:**
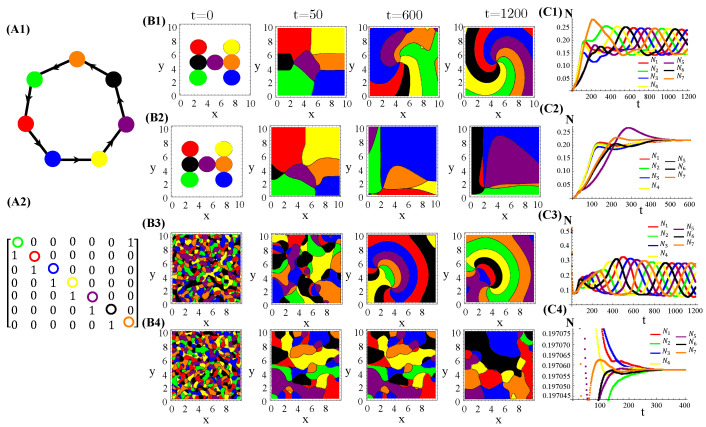
Seven species competing for resources and with cyclic allelochemical suppressions. (**A1**) Schematic illustration of five-species allelochemical warfare. Arrows point from suppressor to suppressed (**A2**). The allelochemical interaction matrix associated to the graph in (**A1**). The initial populations are spatially distributed either regularly in single, disjunct, isolated, and circular patches (**B1**,**B2**) or randomly in adjacent, disjunct but disordered patches (**B3**,**B4**). The corresponding spatial distributions of the species at three distinct times are shown for each initial condition. The different colors indicate the locally dominant species. The evolution in time of the population densities are shown in (**C1**–**C4**). The results in (**B1**,**C1**) and (**B3**,**C3**) refer to response functions dependent on the local concentration of allelochemicals, whereas those in (**B2**,**C2**) and (**B4**,**C4**) are for response only to locally uptaken toxins. The competition and allelochemical traits are the same for all species. The paprameter used are the same as in Figure 1.

## Data Availability

Not applicable.

## Appendix A. Patterns and Boundary Conditions

At weak competition ($\nu_{ij}<1\;\forall i,j$—coexistence regime) nontrivial inhomogeneous (e.g., spiral or concentric) spatial patterns can be self-organized by a RPS suppression game in landscapes subjected to periodic or Newman boundary conditions (Figure A1).

## Appendix B. Patterns and Diffusion

Highly diffusive species and toxins normally induce extinction (even at the coexistence regime) and lead to homogeneous spatial patterns (Figure A2).

## Appendix C. Patterns beyond RPS Allelopathy

RPS-like allelopathic interaction networks involving more than three species can also self-organize inhomogeneous spatial patterns for N=5 (Figure A3), 7 (Figure A4), and 9 (Figure A4) species.

## Appendix D. Patterns and Allelochemical Interaction Networks

Several allelopathic interaction networks for N=3 species, besides the RPS, were tested to investigate their effects on spatial pattern formations (Figure 1(A1)). As one can see in (Figure A6 and Figure A7), homogeneous spatial patterns are generated due to the extinction of two species. In turn, a single extinction leads survivors to self-organize on the landscape in two bands, each one dominated by only one survivor.

## Appendix E. Spatial Correlations between Species and Their Allelochemicals

Spatially organized species distribution patterns are neatly correlated with toxin-spreading patterns on the landscape. According to our results, the species and the allelochemicals they release are in phase, i.e., the biomass peaks and toxins coincide (Figure A9).
